# The role of gender and academic degree on preference for smooth curvature of abstract shapes

**DOI:** 10.7717/peerj.10877

**Published:** 2021-03-09

**Authors:** Letizia Palumbo, Giulia Rampone, Marco Bertamini

**Affiliations:** 1 Department of Psychology, Liverpool Hope University, Liverpool, United Kingdom; 2 Department of Psychology, University of Liverpool, United Kingdom; 3 Department of General Psychology, University of Padova, Padua, Italy

**Keywords:** Visual preference, Curvature, Individual differences, Gender, Academic degree

## Abstract

**Background:**

Preference for smooth contours occurs for a variety of visual stimuli. However, there are individual differences. Openness to experience, a trait associated with aesthetic appreciation, emotional sensitivity and abstract thinking, correlates with this preference. The evaluation of meaningless stimuli entails automatic associations influenced by knowledge, intellectual interests and individual experiences which are diverse. However, it is difficult to capture this variability in studies restricted to Undergraduate students in Psychology with a prevalence of female participants.

**Methods:**

Here we examined preference for curvature with 160 undergraduate students in Psychology, Mathematics, Engineering and Computer Science, balanced for gender. Participants viewed abstract shapes varying for contour (angular vs. curved). The shapes presented variations in Vertices (10, 20, 30) and Concavity (30%, 40%, 50%) to increase complexity. Participants rated how much they liked each shape on a 0 (dislike) to 100 (like) scale. Furthermore, because students in pure Science disciplines present autistic-like traits as measured with the Autism Quotient (AQ), and there is evidence that individuals with autism respond positively to edgy abstract shapes, participants also completed the AQ.

**Results:**

Overall participants preferred curved shapes to angular shapes. We confirmed past research showing that complexity played a key role, with simple shapes with less vertices (10 vertices) being preferred over shapes with larger number of vertices (20 and 30 vertices). Furthermore, simple shapes (10 vertices) were preferred more with more concavities (50%). Importantly, an interaction between academic degree and gender revealed that preference for smooth curvature was stronger for Psychology female participants. Science students scored higher than Psychology students on the AQ. Interestingly, multilevel analyses showed that the variability of AQ traits in the sample did not contribute to this interaction. The results are discussed in relation to theories of preference formation and individual differences.

## Introduction

Humans have the tendency to prefer certain formal properties even in the absence of familiarity or meaning. In particular, there is evidence of preference for regularity, symmetry, smooth curvature, and complexity using abstract shapes or patterns.

In the last decade, there has been growing interest in investigating preference for shape properties. There is evidence suggesting that curved objects elicit pleasure in the observer (Nejad, 2007; Watson et al., 2012) and that curvature is typically associated with positive valence (Palumbo, Ruta & Bertamini, 2015). The idea of a preference for smooth curvature has a long history (for a review and historical perspective see Bertamini et al., 2015). This phenomenon has been documented for a variety of visual stimuli: familiar and unfamiliar objects (Bar & Neta, 2006; Bar & Neta, 2007; Corradi et al., 2019a; Corradi et al., 2019b) geometric or irregular abstract shapes, which prevent familiarity effects (Silvia & Barona, 2009; Bertamini et al., 2015) hand-drawn objects (Bertamini & Sinico, 2021) car design (Leder & Carbon, 2005) and complex interior design environments (Vartanian et al., 2013; Banaei et al., 2017; Ruta et al., 2019). The curvature effect has been replicated across a range of tasks, including explicit forced choice responses (“like”/”no like”), rating scales (Likert scale or visual attention scales—VAS) and selection procedure where participants chose one of two stimuli presented simultaneously (Gómez-Puerto, Munar & Nadal, 2016). However, most of these studies targeted similar populations, such as psychology students, with a prevalence of female participants. This approach has a limitation when it comes to generalising the results. The current study fills this gap by investigating preference for curvature with a large sample of students in different subject areas balanced for gender. We included Psychology undergraduate students to allow a comparison with previous research and we paired them with undergraduate students in Mathematics, Engineering and Computer Science. The interest in individuals studying pure scientific disciplines is due to a body of literature showing differences on personality (i.e., openness to experience) and cognitive skills (i.e., attention to details and analytic thinking) as compared to creative individuals and humanities students (Billington, Baron-Cohen & Wheelwright, 2007; Kaufman et al., 2016; Witkin et al., 1977). This is important because openness to experience and holistic abstract thinking positively correlate with preference for curvature (Cotter et al., 2017).

### The multidimensional nature of preference for curvature

According to an evolutionary perspective, people prefer visual properties that are informative about certain elements in the environment, especially if dangerous, because this would allow programming appropriate responses (Aiken, 1999). Therefore, preference in humans is part of adaptive behaviour. In line with this approach, some authors suggested that people like smooth curvature because sharp angles signal a threat (Bar & Neta, 2006; Bar & Neta, 2007).

Other studies have confirmed that the effect has universal and cross-cultural value by reporting preference for curved objects in great apes (Gomez-Puerto et al., 2016; Gómez-Puerto et al., 2018), in one-week old human babies (Fantz & Miranda, 1975) and in different adult populations living in Spain, Ghana and Mexico (Munar et al., 2015). This supports the existence of a general predisposition in favour of smooth curvature in objects.

Furthermore, preference for smooth curvature occurs in response to automatic semantic associations. Therefore, learning and experience might have boosted this natural propensity. Palumbo, Ruta & Bertamini (2015) using a multidimensional Implicit Association Test (IAT) (Greenwald, McGhee & Schwartz, 1998) found implicit associations of curved shapes with words of positive valance or expressing safety as well as implicit associations of angular shapes with words of negative valance or expressing danger. Interestingly, curved shapes were automatically associated with female names, whereas angular shapes were associated with male names. Therefore, the feminine or masculine connotation of the word was matched with the type of contour of meaningless objects. More recently, this was confirmed by Stroessner et al. (2020) who suggested that these associations extend to masculinity/femininity concepts, gender terms and traits. One explanation is that curved lines visually resemble a female body and from this the association with female names. Another possible explanation for these associations relies on the morphological aspect of curvature. According to Papanek (1995) roundness contributes to the perception of juvenile traits in an object due to an association with a child physiognomy (less body hair, round head, flattened face, and round large eyes). This phenomenon has been called the Baby schema hypothesis (Hildebrandt & Fitzgerald, 1979; Alley, 1981). The manipulation of lines as to increase neoteny features has been used in advertisement and in film industry, especially with cartoons characters. The perception of cuteness evokes feelings of warmth, protectiveness and caretaking behavior (Glocker et al., 2009), which are qualities also attributed to females (Maestripieri & Pelka, 2002).

### Individual differences and preference for smooth curvature

Beside a predisposition to prefer curved objects, fostered by learning, the role of individual characteristics needs consideration. Preference for curvature can be moderated by person factors and individual differences, such for example, art expertise (Silvia & Barona, 2009; Silvia, 2013), or personality traits and cognitive styles (Cotter et al., 2017). Cotter et al. (2017) administered a set of scales, including the Aesthetic Fluency Scale (Silvia, 2007; Silvia, 2013; Swami, 2013), the NEO-Five-Factor Inventory (Costa & McCrae, 1992) and the Types of Intuition Scale (Pretz et al., 2014) and asked participants to rate liking for geometrical and irregular shapes varying for type of contour (curved vs. angular). The results showed a positive correlation between art expertise, openness to experience and preference for curved irregular shapes. The authors’ explanation was linked to the evidence that people high in openness to experience are more imaginative, creative, unconventional, and sensitive to subtle emotions (McCrae & Sutin, 2009; Kaufman, 2013). The results also showed that people who have the tendency to think in abstract terms prefer curved shapes more.

Previous research has showed that preference for smooth curvature is strong and consistent but can be moderated by individual differences. This was recently discussed by Corradi et al. (2019b) who reported an interesting breadth of variation in individual preferences for smooth curvature with real objects and abstract shapes.

Clear differences in preference for curvature have been observed in individuals with autism. Belin et al. (2017) found that individuals with autism spectrum disorders, more than controls, responded positively to edgy abstract stimuli. The authors explained the effect in relation to their atypical emotional responses. According to the DSM V (2013) individuals with autism also show restricted interests, repetitive behaviour, difficulties in imagination and pretence. These individual characteristics deviate from the person factors described by Cotter et al. (2017), which are significant predictors of the preference for curvature.

### The current study

The current study is concerned with the possible factors contributing to individual differences in preference for curvature. A plausible factor that make people different in respect to their preferences is being males or females. Men and women differ in what they perceive as attractive (Djamasbi et al., 2007) and in their aesthetic choices (Johnson & Knapp, 1963; Moss, Gunn & Heller, 2006). These differences are not only due to socialization or gender roles but also to innate factors (Lueptow, Garovich & Lueptow, 1995). We have seen that there is an automatic tendency to associate meaningless shapes with gender categories (Palumbo, Ruta & Bertamini, 2015; Stroessner et al., 2020). The question is whether individual preferences for curvature also depend on gender. This has not been investigated so far. If the association of curved shapes with female categories is due to the curved lines resembling feminine bodily cues, in line with evolutionary theories of sexual selection and mate choice (Jones & Ratterman, 2009), then preference for curvature might be enhanced for male participants. Alternatively, theories of group identification support the idea that we tend to like what resonate with our predispositions and values (Stets & Burke, 2000). If female participants identify more with curved lines, or with the feelings that these might elicit, then there should be an increased preference for curved shapes in females.

When testing participants who are university students, one aspect to consider is the subject of their studies. In general, as much as culture, education and social conventions could play a role on shaping what people like or dislike, also the choice of study, which itself reflects personal predispositions and preference, could play a role. If this is plausible, then the question is whether academic degree contributes to determine individual differences on preference for smooth curvature. As the samples in previous research consisted predominantly of Psychology female students, we did not expect preference for curvature to diminish in this specific group.

Individuals with high autistic traits are predominantly males and have a systemizing cognitive style, which is the drive to understand the rules governing the behaviour of a system, hence allowing control and prediction of the system. In contrast, the empathizing extreme is characterized by understanding and predicting the other person’s mental states, including emotions, and being able to respond appropriately (Baron-Cohen, 2002; Goldenfeld, Baron-Cohen & Wheelwright, 2005; Baron-Cohen, 2016). Male participants, especially those working in the pure sciences, such as engineering and physics, score higher on systemizing (Focquaert et al., 2007). In contrast, the empathizing style is typically more evident in females and in individuals in the humanities (Focquaert et al., 2007). This is reflected in higher scores on the Autism Quotient (AQ) for male participants and students in Science (Baron-Cohen et al., 2001). Based on this evidence and the study by Belin et al. (2017), we measured autistic traits to verify whether our samples of students would differ along the autistic traits continuum and to control the impact of these differences on preference for curvature.

We tested our hypotheses using abstract shapes with a curved or angular contour as implemented in Palumbo & Bertamini (2016) and in Cotter et al. (2017). The task mirrors those used in previous studies where participants rated preference on a 0–100 (0 = dislike to 100 = like) VAS scale (Bertamini et al., 2015; Palumbo & Bertamini, 2016). For this study, we recruited participants from the Psychology, Mathematics, Engineering and Computer Science departments. All participants were students on a BSc programme. It is often the case that samples of Psychology students are predominantly female; we therefore balanced our sample for gender by testing the same number of males and females in each academic degree. From now on we will use the labels Psychology (for students on a Psychology BSc) and Sciences (for students on other BSc programmes, in the area of Mathematics, Engineering and Computer Science).

We further administered the Autism Quotient (AQ) (Baron-Cohen et al., 2001) which provides a continuous measure ranging from low to high autistic-like traits in normative samples.

## Materials & Methods

### Participants

One hundred sixty undergraduate students voluntarily took part in the experiment (age range: 18–44, age mean: 20.34 years, 80 females; 13 left handed). Eighty students were registered in Psychology (age mean: 18.78 SD: 1.14; 40 females; six left handed) and eighty in Science courses (age range: 21.90; SD: 3.82; 40 females; seven left handed). Specific details about the sample along with the AQ scores are reported in Table 1. All participants had normal or corrected-to-normal vision. They provided a written consent for taking part and received course credit for their participation when applicable. The experiment was approved by the Ethics Committee of the University of Liverpool (Approval Ref: IPHS-1415-VA-202) and was conducted in accordance with the British Psychological Society (BPS) Code of Practice.

### Stimuli and apparatus

**Figure 1:**
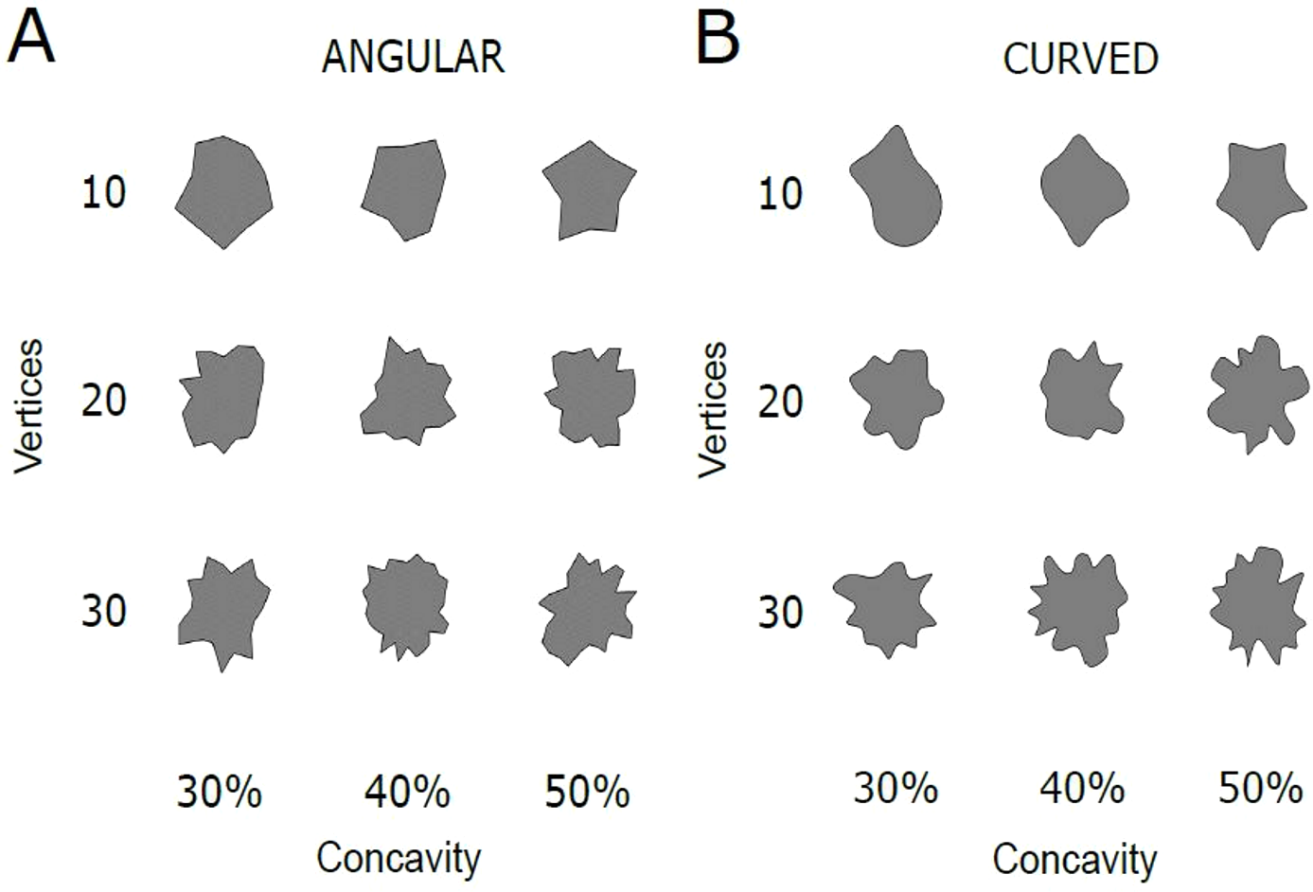
Examples of the stimuli. (A) Angular polygons with variations in Vertices (10, 20, 30) and Concavity (30%, 40%, 50%). (B) Curved polygons with variations in Vertices (10, 20, 30) and Concavity (30%, 40%, 50%).

Stimuli were the same as those used in Palumbo & Bertamini (2016) and consisted of irregular shapes with a black contour of 0.5 px and a grey fill. The outline was curved or angular (straight edges). Stimuli and experiment were created using python and Psychopy (Peirce, 2007). The stimuli were generated starting from polygons that were based on sampling points along a circle, which was chosen as the starting function (see Bertamini et al., 2015). For every point the radius was chosen randomly between 110 (min) and 170 pixels (max). This procedure created unique polygons with a different number of vertices (10, 20 and 30). The number of concavities was controlled within each set of vertices: shapes with 10 vertices contained 3, 4 or 5 concavities; shapes with 20 vertices contained 6, 8 or 9 concavities and shapes with 30 vertices contained 10, 12 or 15 concavities. We can distinguish 3 levels of concavities across the three sets of vertices: 30%, 40% and 50%, as the increase in number of concavities is proportional to the number of vertices (Fig. 1).

**Table 1:** Demographics: mean (±SD) age and Autism Quotient (AQ) for gender and academic degree.

	**AGE**		**AQ**	
	**Females**	**Males**	**Females**	**Males**
Psychology	18.53 (0.82)	19.03 (1.35)	15.1 (6.6)	17.65 (8.1)
Sciences	22.48 (3.51)	21.33 (0.15)	20.5 (6.75)	21.2 (7.6)

For each polygon, a cubic spline generated a curve through the vertices, thus transforming angular vertices in smoothed curves with extrema. To exclude possible effects of memory and familiarity each trial used a different stimulus. However, the stimuli were the same across participants. Distance from the screen was approximately 60 cm. Stimuli were presented on a CTX Vl 950T 19″ CRT monitor (1,600 × 1,200 at 75 Hz).

The software tool used to analyse the data was R-Studio® and the packages functions employed were nlme (specifically the lme() function for the multilevel model; Pinheiro et al., 2020), ez (specifically the ezANOVA() function; Lawrence, 2016) and ggplot2 (for the graphs; Wickham, 2016).

### Procedure

**Figure 2:**
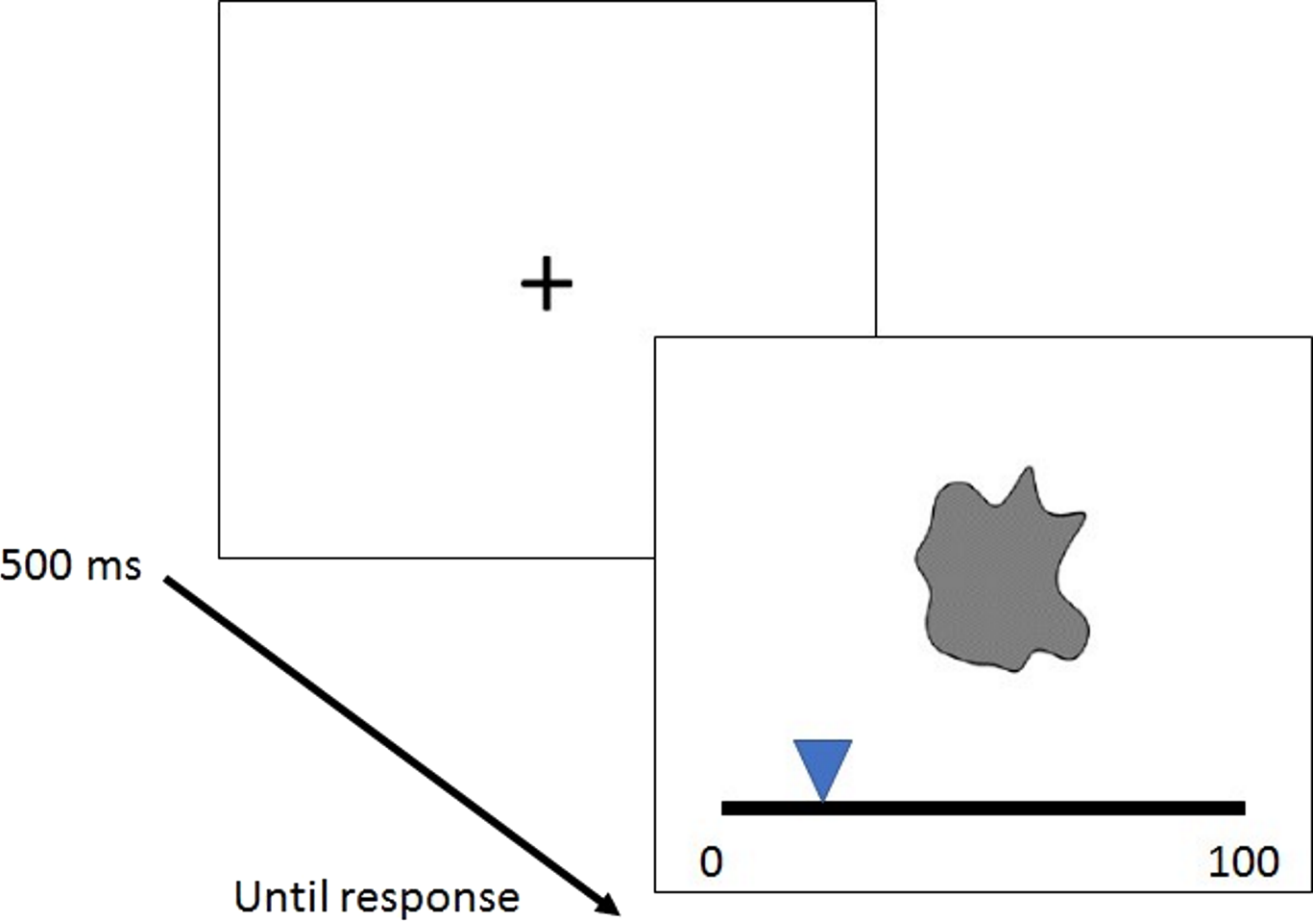
Illustration of a trial. The structure of a trial and the liking rating scale (0 = dislike to 100 = like).

Participants entered their age and gender by indicating whether they are male or female. Following the instructions, each trial started with a fixation cross which was presented at the centre of the screen for 500 ms. Following, the shape was displayed and remained on screen until participants expressed how much they liked the pattern using a rating scale (0 = dislike to 100 = like), see Fig. 2. The experiment started with a practice block (8 trials) followed by 162 experimental trials randomized within one block. The experiment lasted 20 min.

Following the experimental task, participants completed the AQ questionnaire.

The AQ is a 50-items self-administered test (Baron-Cohen et al., 2001) suitable for the general population. The questions cover five different domains associated with the autism spectrum: social skills, communication skills, imagination, attention to detail and attention switching/tolerance of change. Each statement allows the subject to indicate “definitely agree”, “slightly agree”, “slightly disagree” or “definitely disagree”. Scores have been calculated summing the score for each question. A high score (>32) on the AQ test is not by itself diagnostic, but it suggests the presence of significant levels of autistic traits. For the analysis, the scores were centred to the mean, i.e., each individual score was subtracted from the mean of all scores (*M* = 18.6, SD = 7.7). Using mean-centered AQ (AQ_C) scores is more appropriate in this case because we adopted models that also include dummy-coded predictor variables (i.e., form, gender and degree) and interactions with those predictors. The intercept for those predictors is the mean of DV for the reference category (numbered 0, i.e., polygon, male, science). Since AQ scores do not have a meaningful value of 0, centring makes the intercept more interpretable (i.e., mean ratings for polygons for those Science males who scored at the mean level of the AQ spectrum). In this way, the mean value becomes the 0 point for the predictor AQ. Therefore, the slope between the predictor and DV remains unaffected, whilst the interpretation of the intercept changes.

### Experimental design and data analysis

We split the analysis in three parts. The first part includes only factors describing the sample (i.e., non-experimental factors), to explore whether there was a difference in AQ in relation to gender and academic degree (from now on reported as simply “degree” in the statistical models), therefore Psychology versus Science. Second, we tested differences in preference. The dependent variable was the liking rating on the 0–100 scale. The factors related to the stimuli: Contour (angular, smooth) Vertex (10 vs. 20 vs. 30) and Concavity (30%, 40%, 50%). Third, we tested the role of gender and degree on preference (difference between angular and smooth) as the dependent variable. The factors were gender (Male, Female) and degree (Psychology, Science). The fourth and final step in the analysis used linear mixed models to test to what extent individual differences in AQ explain liking ratings for curved and angular shapes.

## Results

### Step 1: Difference in average AQ in relation to gender and degree

**Figure 3:**
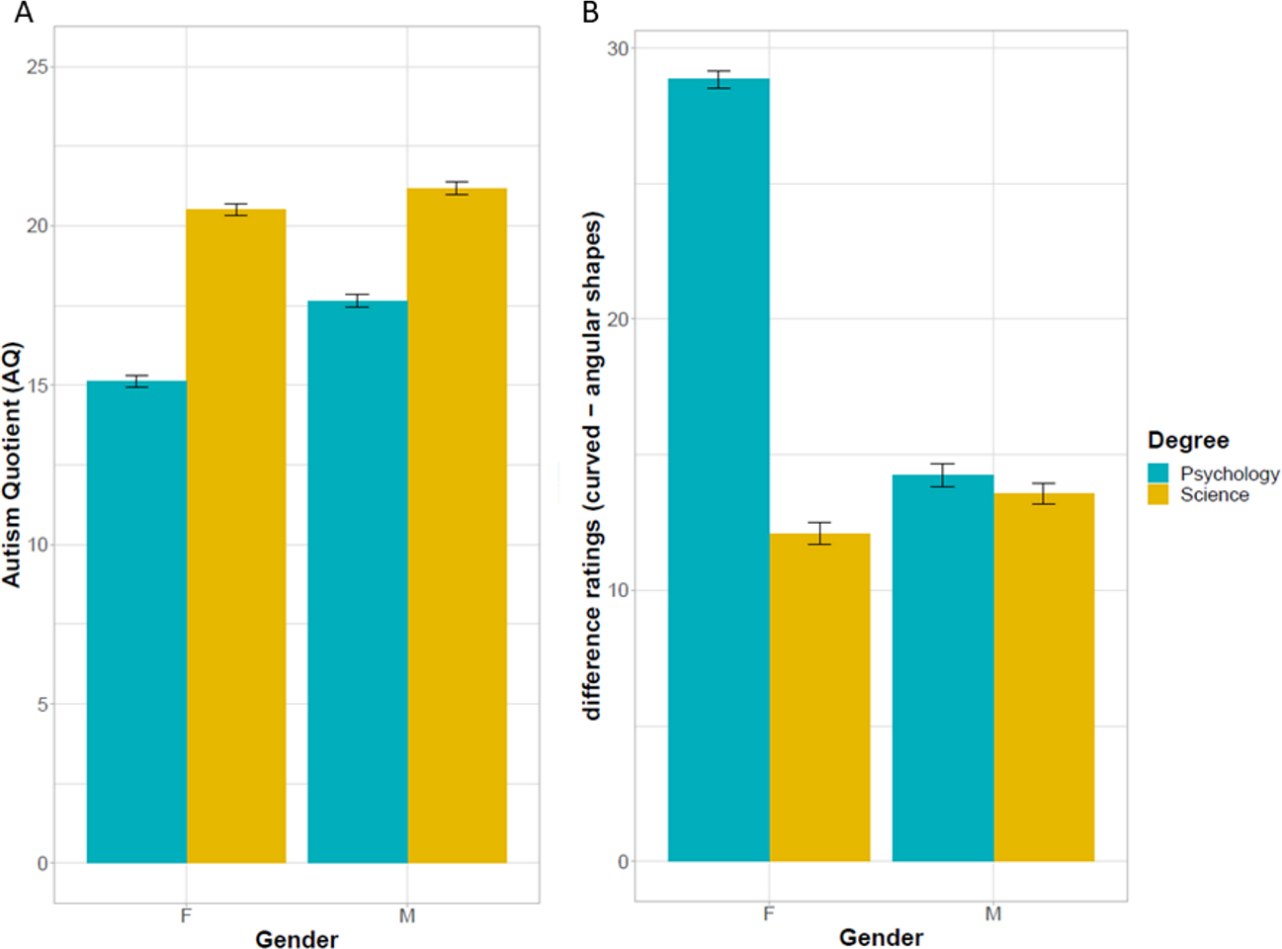
Preference for shapes as a function of gender and academic degree. (A) Autism Quotient (sum score) as a function of gender and degree. Scoring is on a range between 0 (low AQ) to 1 (high AQ). Students in scientific degrees showed greater autism-like traits than psychology students. Males’ AQ was only descriptively greater than females’ AQ. Error bars indicate 95% C.I. (B) Difference ratings for curved shapes–angular shapes as a function of gender (Female, Male) and degree (Psychology, Science). Female psychology students showed a marked preference for curved shapes over angular shapes. This difference in preference was smaller for the other groups.

Table 2 reports the results from the 2 (gender) × 2 (degree) ANOVA on AQ (using AQ_C scores) and shows no difference in AQ scores between males and females. Instead, students of scientific degrees reported significantly higher AQ scores than psychology students. There was no gender × degree interaction (Fig. 3, panel A).

### Step 2: Preference for curvature as a function of number of vertices and concavities

To verify whether our results replicate previous findings (i.e., Palumbo & Bertamini, 2016), we assessed general preference for curvature as a function of number of vertices and concavities. Results are reported in Table 3.

**Table 2:** Results from 2 (gender) × 2 (degree) ANOVA on AQ_C scores.

Predictor	*df*_*Num*_	*df*_*Den*_	*SS*_*Num*_	*SS*_*Den*_	*F*	*p*	${\eta }_{g}^{2}$
(Intercept)	1	156	0.00	8531.25	0.00	1.000	.00
Gender	1	156	102.40	8531.25	1.87	.173	.01
Degree	1	156	792.10	8531.25	14.48	<.001	.08
Gender × degree	1	156	34.23	8531.25	0.63	.430	.00

**Notes.**

*df*_*Num*_ indicates degrees of freedom numerator. *df*_*Den*_ indicates degrees of freedom denominator. *SS*_*Num*_ indicates sum of squares numerator. *SS*_*Den*_ indicates sum of squares denominator. ${\eta }_{g}^{2}$ indicates generalized eta-squared.

**Figure 4:**
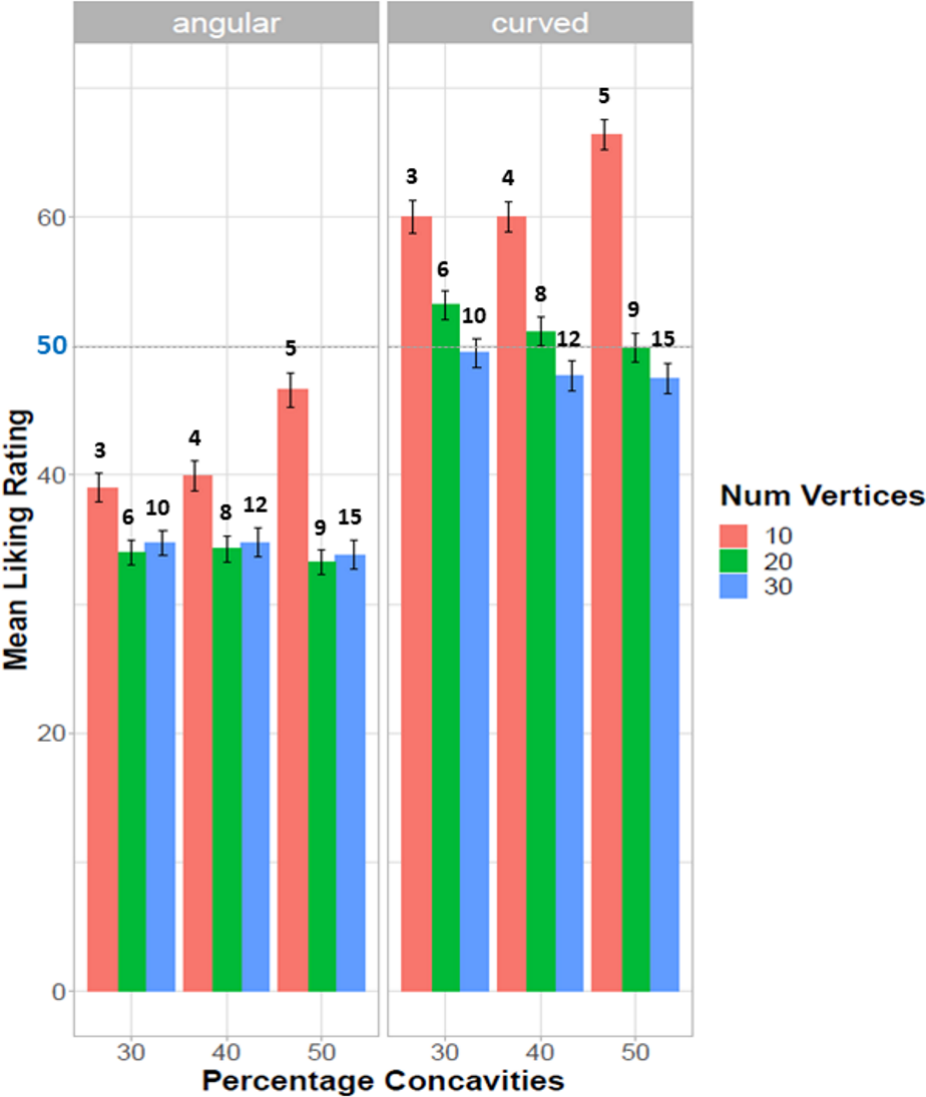
Preference for curvature as a function of number of vertices and proportion concavities. Mean liking rating (*y* axis) as a function of proportion of concavities (30% 40% 50%; *x* axis) and number of vertices (10, 20, 30; red, green, blue colour respectively), displayed separately for angular shapes (left) and curved shapes (right). The actual number of concavities for each shape type is reported above the bars. Each condition shows the average from 1280 trials. Error bars indicate 95% C.I.

Figure 4 illustrates the nature of the significant main effects and interactions (each condition showing the average from 1280 trials; error bars indicating 95% C.I). Ratings were higher for curved shapes than angular shapes. Importantly, if we take 50 as the indifference score (midpoint), curved stimuli tend to be rated above this value, whereas for angular shapes this is less striking, although this tendency is not always systematic as emerges in shapes with 30 vertices. Shapes with 10 vertices were preferred to either 20- or 30-vertices shapes (both for angular and curved shapes). Preference for curved shapes increased as a function of decreasing number of vertices. Among the 10-vertices shapes, shapes with greater percentage of concavities (50%) were preferred (true for both angular and curved shapes). For curved shapes with greater number of vertices (20 and 30) there was a linear increase in preference with decreasing percentage of concavities. Finally, ratings for curved shapes with 30 vertices and 40% and 50% concavities were rated negatively (below the indifference score 50).

**Table 3:** Results from 2 (contour) × 3 (vertices) × 3 (concavities) ANOVA for liking ratings.

Predictor	*df*_*Num*_	*df*_*Den*_	*ϵ*	*SS*_*Num*_	*SS*_*Den*_	*F*	*p*	${\eta }_{g}^{2}$
(Intercept)	1.00	159.00		5913077.35	359057.41	2618.47	.000	.87
Contour	1.00	159.00		212789.85	192293.81	175.95	.000	.20
Vertices	1.13	179.17	0.56	65484.04	176935.21	58.85	.000	.07
Concave	1.34	213.18	0.67	1785.72	29928.03	9.49	.001	.00
Contour × vertices	1.47	233.99	0.74	4138.44	26607.74	24.73	.000	.00
Contour × concave	1.84	292.93	0.92	1112.05	10983.95	16.10	.000	.00
Vertices × concave	3.19	507.64	0.80	8416.78	31521.79	42.46	.000	.01
Form × vertices × concave	3.85	612.85	0.96	360.62	22418.36	2.56	.040	.00

**Notes.**

*df*_*Num*_ indicates degrees of freedom numerator. *df*_*Den*_ indicates degrees of freedom denominator. *ϵ* (epsilon) indicates Greenhouse-Geisser multiplier for degrees of freedom, *p*-values and degrees of freedom in the table incorporate this correction. *SS*_*Num*_ indicates sum of squares numerator. *SS*_*Den*_ indicates sum of squares denominator. ${\eta }_{g}^{2}$ indicates generalized eta-squared.

We chose to analyse percentage of concavities as this allows to compare the same proportions across shapes with different number of vertices. One could consider convexities and concavities themselves (not percentages). This would support the finding that preference peaks for intermediate values. With very few concavities (minimum value 3) the shape is very simple and has no parts, with intermediate values the part structure becomes clear and distinct (the peak for preference is at 5 concavities, this happens for a shape with a total of ten vertices), with a large number of concavities (maximum 15) the shape appears star-like but also spiky and irregular.

### Step 3: Preference for shapes (Difference ratings curved shapes—angular shapes) as a function of gender and degree (degree)

Results from the 2 (gender: female, male) × 2 (degree: psychology, science) ANOVA on the difference preference ratings are displayed in Table 4. Importantly, the interaction gender × degree was significant (*F*(1, 156) = 11.4, *p* = .001, ${\eta }_{g}^{2}=.07$). Preference ratings (difference between angular and smooth) for females Psychology were higher than ratings for females Sciences (*t*(78) = 5.21, *p* < .001), males Psychology (*t*(78) = 4.45, *p* < .001) and males Sciences (*t*(78) = 5.01, *p* < .001). There were no differences in ratings between males Psychology and males Sciences (*t*(78) = 0.19, *p* = .8) and between females Science and males Science (*t*(78) =  − 0.43, *p* = .7); no difference also between males Psychology and females Sciences (*t*(78) = 0.59, *p* = .6) (Fig. 3, panel B).

### Step 4: Taking into account the variability explained by autistic-like traits in liking ratings judgments

In Step 1 analysis we showed differences in AQ scores across groups. It is conceivable to expect that the relationship between preference ratings and the interaction between participant’s gender and degree (see Step 3) might differ for each participant based on their autistic-like traits, in addition to baseline differences across participants and their individual strategies in using the rating scale.

The presented model includes form, gender, degree and AQ_C as fixed factors. The random part of the model includes subject (random intercept; allows the intercept to vary across different subjects) and form (random slope; allows individual differences in the scoring strategy (e.g., tendency to use more central values vs extreme values) used for each level of form to vary within subjects).

**Table 4:** Results from 2 (gender) × 2 (degree) ANOVA for liking ratings.

Predictor	*df*_*Num*_	*df*_*Den*_	*SS*_*Num*_	*SS*_*Den*_	*F*	*p*	${\eta }_{g}^{2}$
(Intercept)	1	156	47286.63	35384.77	208.47	.000	.57
Gender	1	156	1722.66	35384.77	7.59	.007	.05
Degree	1	156	3038.24	35384.77	13.39	.000	.08
Gender × degree	1	156	2586.29	35384.77	11.40	.001	.07

**Notes.**

*df*_*Num*_ indicates degrees of freedom numerator. *df*_*Den*_ indicates degrees of freedom denominator. *SS*_*Num*_ indicates sum of squares numerator. *SS*_*Den*_ indicates sum of squares denominator. ${\eta }_{g}^{2}$ indicates generalized eta-squared.

The model was built up in seven steps described in detail in Supplementary Material 1. This procedure consists on adding each term gradually and comparing each new model to the previous one. Specifically, changes in the -2LL (log-likelihood ratio) across models were used to test whether adding a new term significantly improved the model. This procedure was made to assess the fitness of the presented model and ascertain that it best explains the variability within the data.

Table 5 reports the result of the final model, which showed that the main effect of form and the three-way interaction with gender and degree persisted after taking into account differences in autistic-like traits and the random effects associated to general individual difference and scoring strategies. The interactions form:AQ_C and form:gender:degree:AQ were not significant, suggesting that AQ levels do not explain additional variance in rating curved vs angular shapes.

**Table 5:** Mixed-effect model assessing variance in preference ratings as a function of form, gender, degree and AQ_C whilst controlling for random effects associated to general individual difference and differences in rating strategies for the different levels of form within each individual.

	*b*	*95% CI b*		*SE b*	*df*	*t-value*	*p-value*
(Intercept)	38.2	43.5	47.1	2.07	22872	18.39	<.001
Form	13.5	−9.9	−7.5	2.46	22872	5.50	<.001
Gender	1.29	−2.0	1.6	2.91	152	0.44	0.66
Degree	0.12	−1.7	1.9	2.87	152	0.04	0.97
AQ_C	−0.29	−0.3	0.2	0.26	152	−1.11	0.27
Form:gender	−0.46	0.5	3.0	3.46	22872	−0.13	0.89
Form:degree	0.61	0.8	3.2	3.40	22872	0.18	0.86
Gender:degree	−8.71	−2.1	1.5	4.16	152	−2.09	0.04
Form:AQ_C	0.01	−0.1	0.2	0.31	22872	0.04	0.97
Gender:AQ_C	0.52	−0.1	0.3	0.39	152	1.33	0.18
Degree:AQ_C	0.69	−0.3	0.1	0.35	152	1.94	0.05
Form:gender:degree	14.9	−3.1	−0.6	4.94	22872	3.01	<.001
Form:gender:AQ_C	−0.54	−0.2	0.1	0.46	22872	−1.16	0.25
Form:degree:AQ_C	−0.11	−0.1	0.2	0.42	22872	−0.27	0.79
Gender:degree:AQ_C	−1.15	−0.5	0.0	0.55	152	−2.11	0.04
Form:gender:degree:AQ_C	0.56	−0.2	0.1	0.65	22872	0.86	0.39

**Notes.**

For each fixed-effect table reports *b*-values, 95% Confidence intervals, and standard error; df indicates the degrees of freedom; *t*-values and *p*-values indicate whether the fixed-effect predicts significantly variance in the preference rating.

Some other weaker significant interactions were reported in the model (i.e., interactions gender:degree, degree:AQ_C, gender:degree:AQ_C; Table 5); however, these were not associated with preference ratings for curved over angular shapes.

The fact that AQ_C did not interact with form in our model, might not exclude a role of autistic traits in preference ratings for curvature. We know from the literature that autistic traits are associated to gender and education (Baron-Cohen et al., 2001) and our data confirm a correlation between AQ_C and academic degree (although not between AQ and gender; see Step 1). It is thus conceivable to hypothesize that a model including form, gender and AQ_C, excluding degree, might reveal a significant interaction between form and AQ_C.

Table 6 reports the result of this model, which shows no significant form:AQ interaction but a significant form:gender:AQ interaction. The implication of this result is discussed in the Discussion section.

## Discussion

The literature on the curvature effect suggests that the preference response is strong, but it is not immune from individual variability due to personality and cultural influences (Cotter et al., 2017; Corradi et al., 2019b). It is plausible that other aspects might as well moderate preference for curvature. The evaluative process of meaningless shapes entails automatic associations grounded on knowledge and individual experiences which determine variability in the responses. Most studies investigating visual preference for curvature have used homogenous samples of students in Psychology, which are often predominantly females. This practice has limited our understanding of the phenomenon in relation to individual differences and has prevented the possibility to generalize the results across populations. Therefore, the current study aimed to identify possible individual traits that would moderate visual preference for smooth curvature. In order to do this, we tested samples from two different student populations: students in Psychology and Science including maths, engineering and computer sciences. The experimental design allowed varying the abstract shapes on complexity by increasing the number of vertices and concavities. Visual preference for these stimuli was examined in relation to gender (males vs. females) and academic degree (Psychology vs Sciences). Given the evidence that individuals with autism might prefer edgy abstract shapes (Belin et al., 2017), and that typically students in Science score high on the autistic quotient, we also controlled whether autistic-like traits (as measured with AQ) could contribute to the effect.

**Table 6:** Mixed-effect model assessing variance in preference ratings as a function of form, gender, and AQ_C controlling for random effects associated to general individual difference and differences in rating strategies for the different levels of form within each individual.

	*b*	*95% CI b*		*SE b*	*df*	*t-value*	*p-value*
(Intercept)	37.60	34.8	40.4	1.45	22876	25.92	0.00
form	13.95	10.5	17.4	1.75	22876	7.96	0.00
gender	1.65	2.4	5.7	2.05	156	−0.80	0.42
AQ_C	0.07	−0.3	0.4	0.18	156	0.38	0.71
form:gender	5.96	1.1	10.8	2.48	22876	2.40	0.02
form:AQ_C	−0.06	−0.5	0.4	0.22	22876	−0.26	0.80
gender:AQ_C	0.15	−0.4	0.7	0.27	156	0.55	0.59
form:gender:AQ_C	−0.64	−1.3	0.0	0.32	22876	−1.98	0.05

**Notes.**

For each fixed-effect table reports *b*-values, 95% Confidence intervals, and standard error; df indicates the degrees of freedom; *t*-values and p-values indicate whether the fixed-effect predicts significantly variance in the preference rating.

Our study confirmed a general preference for curved shapes over angular shapes. Specifically, curved shapes were rated more positively (i.e., mean ratings above the mid-point indifference score) than angular ones (i.e., mean ratings below the mid-point indifference score). As in past research (Palumbo & Bertamini, 2016), we observed that complexity played a key role, with simple shapes with less vertices (10 vertices) being preferred over shapes with larger number of vertices (20 and 30 vertices). This is in part explained by the fact that increasing the number of vertices affects the smooth curvature of the contour (i.e., curved shapes with more vertices look spikier). The role of concavities was also interesting: simple shapes (10 vertices) were preferred more with more concavities (50%). Possibly low number of vertices and greater percentage of concavities create the optimal visual configuration, which emphasises the smooth curvature of the shape contour whilst maintaining a high level of interestingness. The combination of number of vertices and number of concavities affected perceived complexity and it seems that intermediate levels of complexity had the highest ratings for preference. This role of complexity is in agreement with the existing literature (Berlyne, 1970; Palumbo & Bertamini, 2016).

Importantly, our data revealed that preference for curvature differed based on academic degree, but this factor interacted with gender. The curvature preference was stronger for female students in Psychology than in the other groups. A multilevel model also showed that this effect persisted independently from the variability on autistic traits, general individual differences across subjects and the different scoring strategies they applied for the different types of shapes.

### What does the interaction of gender with academic degree on preference mean?

There is evidence of implicit links between curvature and feminine attributes (Palumbo, Ruta & Bertamini, 2015; Stroessner et al., 2020). Palumbo, Ruta & Bertamini (2015) showed that gender characteristics, as expressed with female and male names, generate implicit associations with curvature and angularity respectively. The fact that the stimuli were meaningless shapes further suggests that the featural aspect (i.e., contour) of the stimuli is enough to trigger associations. However, whether these attributions would predict a different outcome on the preference for curvature depending on the participants’ gender has never been looked before. Here we found that participants’ gender does play a role but in conjunction with academic degree.

In principle, it is plausible to speculate that, as much as learning social roles and norms has an influence on choices, gender can shape preferences. There is evidence confirming that this is the case. Gender roles and stereotyping has an effect on preferences early in development (Campbell et al., 2000; Serbin et al., 2001; Alexander, Wilcox & Woods, 2009), and on personal choices for different types of objects (Moss, Gunn & Heller, 2006), including works of art (Salkind & Salkind, 1997). The fact that preference for curved shapes was higher in females’ participants studying Psychology supports the view that preference has both social and biological roots. The choice of discipline to study at University reflects person predispositions and preference which in turn could have influenced liking for smooth curvature. Neoteny features enhances cuteness, hence preference for curved shapes. It has been shown that these features elicit caring behaviour especially in females (Glocker et al., 2009). This predisposition might be enhanced in female students, and in Psychology more than in the other subjects.

We measured AQ traits because a previous study found that individuals with autism respond positively to edgy shapes (Belin et al., 2017). Based on previous findings, it was plausible to expect variability on these traits in our student samples. This was confirmed as Science students scored higher than Psychology students on the AQ. Furthermore, autistic traits show different patterns in respect to individual characteristics, including cognitive styles (i.e., deficits in holistic abstract thinking) and negative correlations with openness to experience, which moderate the curvature effect (Happé, 1999; Fortenberry, Grist & McCord, 2011; Schwartzman, Wood & Kapp, 2016).

The multilevel analysis confirmed that gender and academic degree played a major role and showed that AQ traits did not explain additional variability in preferences responses for curved and angular shapes. Given the correlation between AQ traits and degree, a control model, which included AQ but not degree, was calculated. It showed a significant three-way interaction between form, gender and AQ.

These results combined suggest that preference for abstract curvature is likely to be influenced by individual differences related to gender and education, which may be partly explained by differences specific to autistic traits.

One take-home message is that a sample of female psychology students, typical of most studies in partly visual preference for curvature, cannot be considered as representative of the general population. This is in line with recent research showing limitations in any generalisation of results from students to the general public (Hanel & Vione, 2016). Future studies should investigate the role of gender identity in conjunction with education on preference choices and evaluations.

## Conclusion

In conclusion, the current study showed that liking evaluations for abstract shapes are greatly influenced by the contour line as a first parameter and then by complexity. Importantly, our results suggest that individual differences based on gender might play a key role in preference formation although in interaction with choices of study in higher education. Further, this is independent from the presence of autistic like traits, as measured with AQ. The intertwine of academic degree with gender of the participants reveals the importance of person predispositions in preference formation and possibly top-down influences related to gender role differences. Overall, the current study highlights the multifactorial nature of visual preference for curvature and puts forward the necessity to extend the empirical research on visual preference to more heterogeneous samples to clarify the role of individual differences.

##  Supplemental Information

10.7717/peerj.10877/supp-1 Supplemental Information 1 Supplemental tables

10.7717/peerj.10877/supp-2 Supplemental Information 2 Dataset with raw data from 160 participants

## Additional Information and Declarations

### Data Availability

The following information was supplied regarding data availability: Raw data is available in the Supplemental Files.
