# Supplementary material for: The role of gender and academic degree on preference for smooth curvature of abstract shapes
Source: PeerJ. 2021 Mar 9;9:e10877. doi: 10.7717/peerj.10877 (PMC7953868; doi:10.7717/peerj.10877)
Supplement: Supplemental Information 1 [file peerj-09-10877-s001.docx]

**Steps conducted to build up the mixed effects model testing the variability explained by autistic-like traits in liking ratings judgments.**

The presented model assesses whether the interaction between form, participant’s gender and academic degree of study might differ for each individual, based on their autistic-like traits. The model takes into account baseline differences across individuals and differences in rating strategy for each shape type within individuals.

The structure of the model was decided a priori. However, we built it up in several steps by adding each term gradually and comparing each new model to the previous one. This procedure was made to assess the fitness of the presented model and ascertain that it best explains the variability within the data. Specifically, changes in the -2LL (log-likelihood ratio) across models were used to test whether adding a new term significantly improved the model.

We ran seven models using the maximum likelihood method (Twisk, 2006). The models were run on the full dataset containing all trials (i.e. 23040 trials).

The first baseline model includes only the intercept (preference ratings predicted from only the intercept) (Table 1).

**Table 1. Output of the first baseline model including only the intercept**

|  | *b* | *95% CI b* | | *SE b* | *t-value* | *p-value* |
| --- | --- | --- | --- | --- | --- | --- |
| (Intercept) | 45.3 | 45.0 | 45.6 | 0.2 | 276.1 | <.001 |

*Note.* For each fixed-effect table reports *b-values, 95% Confidence intervals*, and *standard error*; *t-values* and *p-values* indicate whether the fixed-effect predicts significantly variance in the preference rating.

Next we fit a model which allows the intercept to vary across different subjects (subject is the first random factor for this model).

**Table 2. Output of the second model introducing *subject* as random intercept**

|  | *b* | *95% CI b* | | *SE b* | *df* | *t-value* | *p-value* |
| --- | --- | --- | --- | --- | --- | --- | --- |
| (Intercept) | 45.35 | 43.6 | 47.0 | 0.9 | 22880 | 51.3 | <.001 |

*Note.* For each fixed-effect table reports *b-values, 95% Confidence intervals*, and *standard error; df* indicates the degrees of freedom; *t-values* and *p-values* indicate whether the fixed-effect predicts significantly variance in the preference rating.

The comparison between these two models is important to ascertain whether there is baseline variation across subjects. Table 8 reports the comparisons of the change in -2LL (log-likelihood ratio) across models, the degrees of freedom for each model and the chi-square distribution and its p-value. The fit of the model improved significantly after modelling the variability in intercepts (model 1 vs model 2; SD= 10.84, 95% CI 9.68 – 12.14; χ^2^ (1) = 4437.4, p <.0001)

In three following steps, we added gradually the predictors form (Table 3), gender (Table 4), degree (Table 5) and AQ_C (Table 6) as fixed terms.

**Table 3. Output of the thrid model introducing form as fixed factor (*subject* as random intercept)**

|  | *b* | *95% CI b* | | *SE b* | *df* | *t-value* | *p-value* |
| --- | --- | --- | --- | --- | --- | --- | --- |
| (Intercept) | 36.7 | 35.0 | 38.5 | 0.9 | 22879 | 41.1 | <.001 |
| form | 17.2 | 16.7 | 17.7 | 0.3 | 22879 | 63.3 | <.001 |

*Note.* For each fixed-effect table reports *b-values, 95% Confidence intervals*, and *standard error; df* indicates the degrees of freedom; *t-values* and *p-values* indicate whether the fixed-effect predicts significantly variance in the preference rating.

**Table 4. Output of the fourth model introducing gender as fixed factor (*subject* as random intercept)**

|  | *b* | *95% CI b* | | *SE b* | *df* | *t-value* | *p-value* |
| --- | --- | --- | --- | --- | --- | --- | --- |
| (Intercept) | 37.7 | 35.2 | 40.1 | 1.3 | 22878 | 29.9 | <.001 |
| form | 13.9 | 13.2 | 14.7 | 0.4 | 22878 | 36.3 | <.001 |
| gender | 1.9 | 1.6 | 5.4 | 1.8 | 158 | -1.1 | 0.3 |
| form:gender | 6.6 | 5.5 | 7.6 | 0.5 | 22878 | 12.1 | <.001 |

*Note.* For each fixed-effect table reports *b-values, 95% Confidence intervals*, and *standard error; df* indicates the degrees of freedom; *t-values* and *p-values* indicate whether the fixed-effect predicts significantly variance in the preference rating.

**Table 5. Output of the fifth model introducing degree as fixed factor (*subject* as random intercept)**

|  | *b* | *95% CI b* | | *SE b* | *df* | *t-value* | *p-value* |
| --- | --- | --- | --- | --- | --- | --- | --- |
| (Intercept) | 37.4 | 33.9 | 40.9 | 1.8 | 22876 | 21.0 | <.001 |
| form | 13.6 | 12.5 | 14.6 | 0.5 | 22876 | 25.3 | <.001 |
| gender | 2.5 | -2.5 | 7.4 | 2.5 | 156 | 1.0 | 0.3 |
| degree | 0.5 | -4.5 | 5.4 | 2.5 | 156 | 0.2 | 0.9 |
| form:gender | -1.5 | -3.0 | 0.0 | 0.8 | 22876 | -2.0 | 0.1 |
| form:degree | 0.7 | -0.8 | 2.2 | 0.8 | 22876 | 0.9 | 0.4 |
| gender:degree | -8.7 | -15.7 | -1.6 | 3.6 | 156 | -2.4 | <.001 |
| form:gender:degree | 16.1 | 14.0 | 18.2 | 1.1 | 22876 | 15.0 | <.001 |

*Note.* For each fixed-effect table reports *b-values, 95% Confidence intervals*, and *standard error; df* indicates the degrees of freedom; *t-values* and *p-values* indicate whether the fixed-effect predicts significantly variance in the preference rating.

**Table 6. Output of the sixth model introducing AQ_C as fixed factor (*subject* as random intercept)**

|  | *b* | *95% CI b* | | *SE b* | *df* | *t-value* | *p-value* |
| --- | --- | --- | --- | --- | --- | --- | --- |
| (Intercept) | 38.2 | 34.5 | 41.8 | 1.9 | 22872 | 20.6 | <.001 |
| form | 13.5 | 12.4 | 14.6 | 0.6 | 22872 | 24.0 | <.001 |
| gender | 1.3 | -3.8 | 6.4 | 2.6 | 152 | 0.5 | 0.6 |
| degree | 0.1 | -4.9 | 5.2 | 2.6 | 152 | 0.0 | 1.0 |
| AQ_C | -0.3 | -0.7 | 0.2 | 0.2 | 152 | -1.2 | 0.2 |
| form:gender | -0.5 | -2.0 | 1.1 | 0.8 | 22872 | -0.6 | 0.6 |
| form:degree | 0.6 | -0.9 | 2.1 | 0.8 | 22872 | 0.8 | 0.4 |
| gender:degree | -8.7 | -16.0 | -1.4 | 3.7 | 152 | -2.3 | <.001 |
| form:AQ_C | 0.0 | -0.1 | 0.1 | 0.1 | 22872 | 0.2 | 0.9 |
| gender:AQ_C | 0.5 | -0.2 | 1.2 | 0.3 | 152 | 1.5 | 0.1 |
| degree:AQ_C | 0.7 | 0.1 | 1.3 | 0.3 | 152 | 2.2 | <.001 |
| form:gender:degree | 14.9 | 12.7 | 17.1 | 1.1 | 22872 | 13.1 | <.001 |
| form:gender:AQ_C | -0.5 | -0.7 | -0.3 | 0.1 | 22872 | -5.1 | <.001 |
| form:degree:AQ_C | -0.1 | -0.3 | 0.1 | 0.1 | 22872 | -1.2 | 0.2 |
| gender:degree:AQ_C | -1.2 | -2.1 | -0.2 | 0.5 | 152 | -2.4 | <.001 |
| form:gender:degree:AQ_C | 0.6 | 0.3 | 0.8 | 0.1 | 22872 | 3.7 | <.001 |

*Note.* For each fixed-effect table reports *b-values, 95% Confidence intervals*, and *standard error; df* indicates the degrees of freedom; *t-values* and *p-values* indicate whether the fixed-effect predicts significantly variance in the preference rating.

The final model introduced *form* as another random term (random slope) in addition to *subject*. This is reported here (Table 7) as well as in the manuscript. Adding form as a random factor (as well as fixed factor) means that scores for each level of form (curved, angular) are nested within participants. In this way if there are individual differences in the strategy used for scoring (e.g. tendency to use more central values vs extreme values) these are taken into account.

Table 8 shows the output of the comparison analysis made on each new model in the order the models were built. It shows that every new model reduces -2LL significantly, meaning an improved fitness of the model in explaining the variability in the data.

**Table 8. Outputs of the comparison analyses conducted on each new model vs the previous model to assess their fitness**

|  | Model | *df* | | *AIC* | *BIC* | *logLik* | *Test* | *L.Ratio* | *p-value* |
| --- | --- | --- | --- | --- | --- | --- | --- | --- | --- |
| 1 | Intercept Only | 2 | | 213534.5 | 213550.6 | -106765.3 |  |  |  |
| 2 | Random Intercept Only (subject) | | 3 | 209099.2 | 209123.3 | -104546.6 | 1 vs 2 | 4437.4 | <.0001 |
| 3 | Form fixed-effect | 4 | | 205406.3 | 205438.5 | -102699.2 | 2 vs 3 | 3694.8 | <.0001 |
| 4 | Gender fixed-effect | 6 | | 205263.2 | 205311.4 | -102625.6 | 3 vs 4 | 147.2 | <.0001 |
| 5 | Degree fixed-effect | 10 | | 204785.9 | 204866.4 | -102383.0 | 4 vs 5 | 485.2 | <.0001 |
| 6 | AQ_C fixed-effect | 18 | | 204749.5 | 204894.3 | -102356.8 | 5 vs 6 | 52.4 | <.0001 |
| 7 | Random Slope (form) | 20 | | 202138.4 | 202299.3 | -101049.2 | 6 vs 7 | \| 2615.1 \| <.0001 \| \| --- \| --- \| | <.0001 |

*Note.* *df* indicates degrees of freedom for the model. *AIC* indicates Akaike information criterion. *BIC* indicates Bayesian information criterion. AIC and BIC are measures of fit of the model; a decrease in these values from a model to another model indicates the fit is improving, an increase in these values indicates a worse fit. *logLik* indicates the log-likelihood. *L.Ratio* is the likelihood ratio.

Adding *form* as random slope did benefit the model (Table 4; SD = 7.2, CI 6.4 – 0.16; χ2 (2) = 2615.1, p <.001), although this caused the interactions with AQ_C to disappear.

The slopes and intercepts were not significantly correlated (r= - 0.13, CI -0.4 – 0.2).
